# Bovine Mastitis *Vis a Vis Staphylococcus* spp. Mediated Antimicrobial Resistance at Animal-Human Interface in Organized and Unorganized Dairy Sectors: A Study from Two Indian States

**DOI:** 10.3390/antibiotics15030256

**Published:** 2026-03-02

**Authors:** Devi Murugesan, Bibek R. Shome, Nimita Venugopal, Praveen K. A. Muninarayanaswamy, Rituparna Tewari, Pavan K. Nagaraja, Nagalingam Mohandoss, Somy Skariah, Yogisharadhya Revanaiah, Snigdha M. Maharana, Gandu Shanmugam, Shivasharanappa Nayakwadi, Mohan Papanna, Rajeswari Shome

**Affiliations:** 1 Indian Council of Agricultural Research-National Institute of Veterinary Epidemiology and Disease Informatics (ICAR-NIVEDI), Yelahanka, Bengaluru 560119, India; devisanna@gmail.com (D.M.); brshome@gmail.com (B.R.S.); nimitacv@gmail.com (N.V.); praveenkumargwda@gmail.com (P.K.A.M.); tewarirituparna@gmail.com (R.T.); pavankalyangowda1998@gmail.com (P.K.N.); nagar75@gmail.com (N.M.); somyskaria@gmail.com (S.S.); dryogish.vet@gmail.com (Y.R.); madhabamaharana192@gmail.com (S.M.M.); gshanmugam846@gmail.com (G.S.); drshivasharan@gmail.com (S.N.); 2 Crestwood Medical Center, Huntsville, AL 35801, USA

**Keywords:** bovine mastitis, *Staphylococcus* spp., antimicrobial resistance, dairy sectors, India

## Abstract

A comparative cross-sectional study was undertaken in organized and unorganized dairy sectors to evaluate the prevalence of bovine mastitis and the antibiotic resistance in *Staphylococcus* spp. of dairy animals and their associated personnel. A total of 391 households (HH) consisting of 211 and 180 HHs from organized and unorganized sectors, respectively, were selected based on 30-cluster sampling methodology in southern and northeastern regions of India. From 391 HHs, a total of 1920 milking cows (organized dairy—533; unorganized dairy—1387) were screened for subclinical and clinical mastitis by the California Mastitis Test (CMT). Out of 1920 milk samples, 1002 milk samples, 362 associated personnel hand and nasal swabs, and 27 milking machine swabs were sourced. The samples were subjected to *Staphylococcus* spp. by isolation and identification by multiplex polymerase chain reactions (mPCRs) and antibiotic sensitivity testing (ABST) to determine antimicrobial resistance (AMR) profiles. CMT results showed high mastitis prevalence (54.65%) in unorganized farms compared to organized ones (45.78%), with a significant association of mastitis to dairy sectors (*p* = 0.0004). On speciation, *S. aureus* isolates were comparatively less than those of coagulase-negative staphylococci (CoNS) (3.5% and 7.7%, respectively) in the organized dairy sector, and the same was recorded for the unorganized dairy sector (0.85% and 13.19%, respectively). In both the dairy sectors, the highest antibiotic resistance for *Staphylococcus* spp. was observed against the β-lactams (penicillins and cephalosporins) group (71.36% and 76.59%) and the lowest for nitrofurans (3.5% and 3%), oxazolidines (0.7% and 5.1%), and rifamycin (0.7% and 5.1%), respectively. In both the sectors, human isolates had comparatively high *mecA* positives (15.70% and 15.96%) compared to the animal isolates (8.36% and 12.94%). Based on mPCR, a smaller number of methicillin-resistant *S. aureus* (MRSA) isolates (3.95%) than methicillin-resistant coagulase-negative Staphylococci (MRCoNS) was detected in milk samples (6.05%), and the same was observed for associated personnel samples (MRCoNS (14.63%) compared to MRSA (1.05%)). In four HHs, *mecA* positives were detected in both animal and human samples, and this highlights the transmission dynamics of *mecA* between animals and humans in households. The resistance of *Staphylococcus* spp. to β-lactams highlights the need for cautious antibiotic use to prevent AMR.

## 1. Introduction

Mastitis, an inflammatory condition of the mammary gland, poses multifaceted repercussions on milk production, farm economics, and public health problems due to potential zoonotic infections [1]. Mastitis is also one of the greatest constraints to milk production globally and it is categorized into clinical (CM) and sub-clinical (SCM). The diagnosis of CM in cattle is comparatively easy, with visible signs like reddened and swollen udders that can lead to fatal outcomes in severe cases [2], whereas the diagnosis of SCM is problematic, as milk appears normal with no visible signs in the udder. Both CM and SCM can be diagnosed through measurement of the somatic cell counts (SCCs) or by the California Mastitis Test (CMT) [3]. The CMT is a very simple, cost-effective, field-based and fast screening test useful for the detection of both SCM and CM cattle [4]. Studies conducted in different parts of Sub-Saharan Africa (SSA) revealed that SCM and CM prevalence exceeding 50% and were perceived to be a threat for the small-scale producers [5].

Mastitis occurs as an immune response to bacterial invasion as a result of various chemical, mechanical, or thermal injuries to the udder transmitted through contact with contaminated materials in housing, bedding, milking hand, milking machine, etc. Although over 200 microorganisms have been described as potential causative agents, only a few species of Staphylococci, Streptococci, and coliforms are of major economic and epidemiological importance [6]. *Staphylococcus aureus*, a coagulase-positive species which produces a series of enzymes and toxins, has been frequently implicated in the etiology of a series of infections and intoxications in animals and humans, whereas coagulase-negative staphylococci (CoNS), representing the majority of species, have been considered to be saprophytic or rarely pathogenic [7]. In recent years, research has shown that CoNS have emerged as the primary pathogens responsible for SCM and CM in numerous countries [8,9].

Antimicrobial therapy is the most widely used treatment for mastitis control. The widespread use of antibiotics for the control of mastitis greatly increases the risk of transmitting antibiotic resistance to consumers, and this possibility is constantly in the attention of public health authorities [10]. It has been reported that both methicillin-resistant *S. aureus* (MRSA) and methicillin-resistant coagulase-negative Staphylococci (MRCoNS) are the potential causative agents complicating the mastitis treatment [11,12]. The MRSA is an important pathogen that is currently garnering prime attention in the animal health sector due to its risk of transmission to other animals, associated personnel, and into the food chain. A recent report suggests the importance of bacteriological examination of SCM or CM in cattle before starting the treatment to avoid antimicrobial therapy for ensuring prudent use of antimicrobials [13].

Emergence of antibiotic resistance in humans, animals, and the environment is associated with the unnecessary use and misuse of antibiotics, which is contributing to the global emergence of antimicrobial-resistant (AMR) bacteria, a threat to public health and infection control. Currently, India is the world’s leading milk producer and globally ranks fourth in its usage of antibiotics in livestock [14,15,16]. It is estimated that approximately 70 million households (HHs) derive their livelihoods from dairy cattle across India [17]. On these small-scale dairy farms, non-therapeutic or irrational use of antibiotics in lactating cows are uncontrolled and unregulated [18,19].

AMR is an emerging public health issue globally but the problem may be even more serious in developing countries. India has a high burden of infectious diseases, and the uncontrolled access to medicines may lead to higher consumption and inappropriate use of antibiotics, resulting in higher levels of resistance [20,21]. The guidance and supportive intervention strategies at the farm level may have an impact on the reduction in the emergence of AMR. This One Health surveillance study aimed to assess mastitis prevalence and AMR in *Staphylococcus* spp. across two dairy sectors and to evaluate transmission dynamics at the human–animal interface.

## 2. Results

### 2.1. Analysis of Results from the Organized Dairy Sector of Karnataka

**Figure 1:**
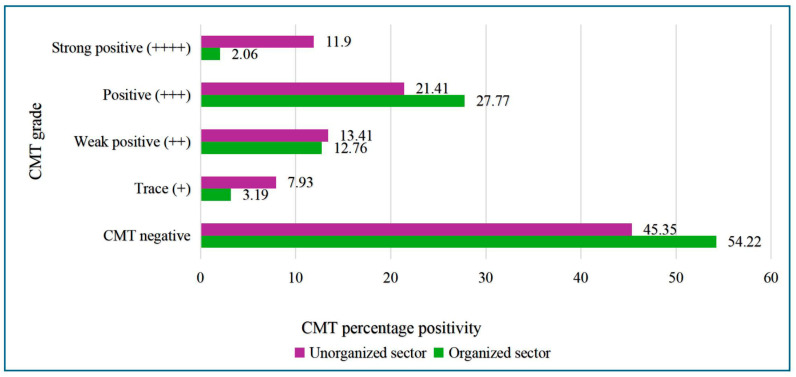
Distribution of CMT positivity between organized and unorganized dairy sectors.

In the organized dairy sector, 211 HHs were visited, having 533 cattle, of which 489 samples were collected, constituting 244 milk samples, 109 each of hand and nasal swabs, and 27 milking machine samples. Overall, CMT positivity of 45.78% was noted in milk samples. Among the four blocks visited, Bengaluru north block had the highest CMT positives (61.79%) and significant difference to CMT positives was observed between the four blocks (*p* = 0.011) (Table 1). The highest number of animals were declared CM cases (27.78%) compared to SCM cases based on CMT results (12.76%) (Figure 1). Quarter-wise prevalence of both CM and SCM mastitis cases was highest in left-hind (56.56%) and right-hind quarters (53.69%), followed by left-fore and right-fore quarters (48.3% each).

Out of 489 samples screened for bacterial isolation, *Staphylococcus* spp. were predominantly recovered from milk samples (92.84%, 454/489), followed by associated personnel hand (53.08%, 241/454), and nasal samples (42.07%, 191/454). The lowest recovery was from milking machine samples (4.85%, 22/454). *S. aureus* isolates were less frequent (3.52%, 16/454) than CoNS (7.7%, 35/454) (Table 2).

**Figure 2:**
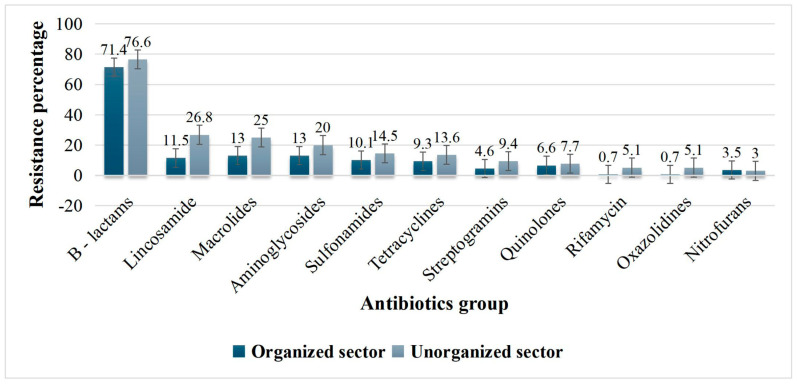
Antibiotic sensitivity profiles across the study sites.

Out of 454 *Staphylococcus* spp. isolates tested for 11 groups of antibiotics by AST, maximum antibiotic resistance was observed against the β-lactam group (71.36%) followed by 13% each for macrolides, aminoglycosides, and 11.45% for lincosamides (Figure 2).

Among 454 *Staphylococcus* spp. isolates, 51 isolates (11.23%) were *mecA*-positive by PCR. Of *mecA*-positive 51 isolates, 16 isolates were of MRSA type and 35 were of MRCoNS (*S. epidermidis*-24, *S. haemolyticus*-4, *S. chromogenes*-3, *S. hominis*-2, and one each of *S. saprophyticus* and *S. sciuri*) (Table 3 and Table 4). The *mecC* genes could not be detected in the isolates. Of the total *mecA*-positive isolates, significant human samples were the highest (15.70%) compared to milk samples (8.36%) (*p* = 0.025). *S. epidermidis* isolates from milking machine swabs showed the *mecA* gene. In milk samples also, the highest numbers of MRSA isolates (5.39%, 13/241) were detected compared to MRCoNS (2.9%, 7/241), whereas MRCoNS were the predominant species in associated personnel (14.14%, 27/191) compared to MRSA isolates (1.57%, 3/191) (Table 5).

### 2.2. Analysis of Results from the Unorganized Dairy Sector of Assam

From the Assam region, a total of 902 samples were sourced from 180 HHs having 1387 cattle from three blocks. The majority of the samples were from milk (758), and 76 and 68 samples were from the associated personnel hand and nasal swabs, respectively. Overall burden of mastitis was very high (54.65%). The significant association was observed for mastitis between organized and unorganized sectors (*p* = 0.0004). Within Assam, Dispur block showed the highest CMT positivity (65.07%), followed by Guwahati (55.09%) and Sonapur blocks (49.18%), with a significant association with mastitis (*p* = 0.0003) (Table 2). Mastitis was highest in the left- and right-hind quarters (56.56 and 53.69%), and both left- and right-fore quarters had 48.52% each CMT positivity. The CM cases were high (21.41%) compared to SCM cases (13.41%) in the Assam region, too (Figure 1).

Out of 902 samples subjected to bacterial isolation, 26.05% (235/902) isolates were of *Staphylococcus* spp. The majority of these isolates were from milk samples (59.15%, 139/235) followed by associated personnel hand (17.02%, 40/235) and nasal samples (23.82%, 56/235). On speciation by mPCR, *S. aureus* isolates were few compared to CoNS in overall samples (0.85% and 13.19%).

Out of 235 *Staphylococcus* spp. tested by AST, maximum antibiotic resistance was observed against the β-lactam group (76.59%), similar to that of the organized sector. The next highest resistance was observed against lincosamides (26.8%), macrolides (25.1%), and aminoglycosides (20%). A similar resistance pattern was observed in the *Staphylococcus* spp. isolated from the organized sector (Figure 2).

A total of 33 isolates among 235 *Staphylococcus* spp. (14.04%) were positive for the *mecA* gene, of which only two isolates were *S*. *aureus* (MRSA) and 31 were MRCoNS isolates (*S. epidermidis*-18, *S. haemolyticus*-11, and *S. scuiri*-2). Comparatively, a smaller number of *mecA*-resistant isolates were detected in animals (12.94%, 18/139) compared to humans (15.62%, 15/96), with a non-significant association between human and animal sectors (Table 3 and Table 4). In none of the HHs were *mecA* positives detected at the human and animal interface, unlike in the organized sector. Within milk samples, predominantly MRCoNS were found in high numbers (11.51%, 16/139) compared to MRSA (1.44%, 2/139), whereas all were MRCoNS in associated personnel samples (Table 5).

Overall results showed a significant association for mastitis between the two dairy sectors, as CMT positives were high in the unorganized dairy sector (54.65%) compared to the organized dairy sector (45.78%). Very high mastitis in the left- and right-hind quarters and the least in the left- and right-fore quarters was a common observation noted with respect to teat predisposition in both the dairy sectors. With respect to *Staphylococcus* spp. isolation, the highest recovery was from milk samples of the organized dairy sector (92.84%), compared to the unorganized dairy sector (26.05%).

**Figure 3:**
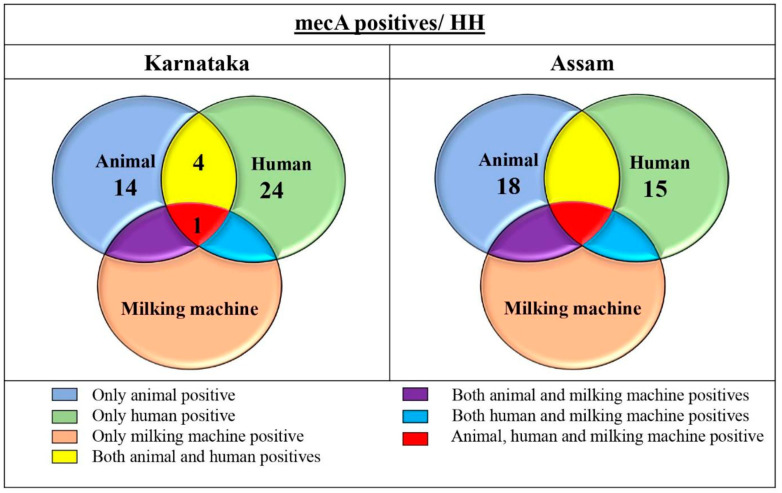
Venn diagram representing *mecA*-positive *Staph.* spp. in both sectors.

Among species, CoNS were the dominant species over *S. aureus* in both the sectors. By AST, the same pattern of resistant profiles was observed with maximum antibiotic resistance to the β-lactam group, followed by lincosamides, macrolides, aminoglycosides, and the least to enrofloxacin. In the organized sector, human samples had more *mecA* isolates (15.70%) compared to animal isolates (8.36%), with a significant difference (*p* = 0.025). Even in the unorganized sector, more *mecA* isolates were detected in humans (15.96%) than from animals (12.94%). In four HHs, *Staphylococcus* spp. harboring *mecA* was observed in both animal and human samples, and in another HH, *mecA* was detected in *S. epidermidis* in human, milk, and milking machine samples (Figure 3).

## 3. Discussion

Mastitis is one of the most important diseases in dairy animals across the globe. The economic losses incurred due to clinical and subclinical mastitis include treatment costs, reduced milk production, and poor quality of milk [22]. Although a wide spectrum of bacterial species are implicated in mastitis, only a few species of Staphylococci, Streptococci, and coliforms are of economical and epidemiological importance [23]. In the present study, mastitis prevalence and AMR mediated through the major mastitis-causing organism, *Staphylococcus* spp., is assessed in two different sectors of dairy farming.

The CMT is an efficient indicator of udder health and a tool for the detection of mastitis. Increased somatic cell counts (SCCs), particularly SCC > 200,000 cells/mL, is a cut-off point recommended for diagnosis of SCM with maximum sensitivity and specificity and minimal diagnostic error [24]. Reports suggest the reliability of CMT up to 85.69% for its ability to correctly identify the udder health status of cows [4,24]. Considering the field applicability of the test on a large number of milk samples, we performed CMT in the current study. The highest prevalence of mastitis was noted in the unorganized dairy sector (54.65%) compared to the organized dairy sector (45.78%). This may be due to overcrowding of 30–50 animals housed in a single shed with limited space and poor hygiene. Different reports have collectively reported a prevalence of SCM ranging from 9% to 86% across 21 regions/states in the country [23].The huge burden of SCM combined with consumption of raw milk (a religious belief in various parts of India) can have adverse public health implications [25].

In the current study, in both the dairy sectors, a total of 1920 animals, constituting 7680 quarters, were assessed for mastitis, of which left-hind and right-hind quarters showed the highest mastitis prevalence in both organized (56.56% and 53.69%, respectively) and unorganized sectors (63.09% and 57.64%). The reasons include the larger size of the hind quarters and greater chances of environmental and fecal contamination owing to their anatomical location [26].

Based on CMT grading, animals were categorized as healthy, SCM, and CM. The high number of CM cases recorded (21.41%) compared to SCM cases (13.41%) in the study represents the burden of mastitis on the dairy sector. The estimated annual economic loss due to overall mastitis in India was US$ 98,228 million (7165.51 crore Indian Rupees) as per reported studies [27]. The high percentage of CM cases recorded in the study clearly indicates a lack of on-farm testing and appropriate timely interventions, which has allowed SCM to progress to CM. In the focus group discussions, farmers have identified mastitis as major animal health challenge, followed by foot and mouth disease (FMD) in the regions.

In AST, in *Staphylococcus* spp. resistance for β-lactams (Penicillins and cephalosporins), aminoglycosides (Gentamicin), macrolides (Erythromycin), lincosamides (Clindamycin), quinolones (Levofloxacin), streptogramins, tetracyclines, etc., was observed in a descending order. Recent studies have shown that the most common indication for using antibiotics in cattle is mastitis, and the preferred antibiotics include β-lactams and streptomycin [17]. Probably, long-term usage of these recommended antibiotics for mastitis treatment has resulted in high resistance, as observed in our study in both sectors [28]. Antibiotic residues in 21% of milk samples, of which 7.1% and 5.52% of samples had maximum residual limits (MRLs) and multidrug residues (MDRs), respectively, from the organized dairy sector of Karnataka correlated with resistance profiles observed in *Staphylococcus* spp. [23]. The contribution of antimicrobial use (AMU) to AMR has become a concern due to actual or predicted adverse effects on human and veterinary health [28,29].

Mastitis is considered to be a complex disease to control because several bacteria are capable of infecting and producing the disease in dairy cattle. *Staphylococcus* spp. is the major causative organism in mastitis and is highly contagious. In our study, the majority (72.4%) of *Staphylococcus* spp. recovered were from milk samples of the organized sector, compared to the unorganized sector (30%), though mastitis percentage was high in the latter region. The inability to isolate bacteria from milk samples may be due to elevated SCC, altered pH, a low concentration of pathogenic microorganisms, intermittent shedding of pathogens, the intracellular location of pathogens, the presence of growth inhibitors, or the spontaneous elimination of the infection from the udder [30]. The other reason for the low percentage of *Staphylococcus* spp. isolation is difficulties encountered during primary isolation carried out in the field unit of Assam, India.

In *Staphylococcus* spp., methicillin resistance primarily stems from altered penicillin binding protein (PBP2a), which exhibits minimal affinity for β-lactam antibiotics. The *mecA* gene regulates PBP2a, and its identification among staphylococcal isolates is considered the gold standard for detecting methicillin resistance. Therefore, we isolated *Staphylococcus* spp. on MSA and detected the *mecA* gene using PCR. The percentage of methicillin-resistant Staphylococci (MRS) was insignificantly high in the unorganized (12.94%) compared to the organized dairy sector (8.36%). The greater *Staphylococcus* spp. diversity in Karnataka observed in the study was attributed to quick transport of the samples to the laboratory, consistent isolation through established protocols, and further confirmation by mPCR. However, the rate of *mecA*-positive isolates identified in our study was comparatively less than the other reported studies, probably due to the cluster sampling approach [31]. In both dairy sectors, MRSA were notably high from human samples (15%).

Both MRSA and, to a lesser extent, MRCoNS have been increasingly reported in mastitis with potential transmission between animals and humans [11,12]. The CoNS are considered less-known pathogens of bovine mastitis; however, many studies recently have shown the importance of CoNS infection in the bovine mammary gland. Several studies have reported CoNS from mastitis samples, especially during first lactation and unbred heifers [7,8]. In European countries, the prevalence rate of bovine MRSA ranges from 0.4 to 17% [11,32], and among Asian countries, India and China reported the prevalence rate of bovine MRSA to be 13 and 48%, respectively [33,34]. High prevalence of CoNS may be attributed to the widespread distribution of the organism within the mammary gland, udder teats, and farm environment. Using species-specific mPCR, it was observed that *mecA*-positive *S. aureus* (MRSA) isolates were less (24%) compared to MRCoNS (76%) in the organized dairy sector, underscoring the importance of CoNS as a reservoir of methicillin resistance [35]. Methicillin resistance, likely influenced by the presence of resistance genes, has been observed as per the recent reports in Indian dairy sectors [36].

Accurate species identification in mastitis is critical for therapy and epidemiological surveillance. The mPCR assays have proven to be effective in differentiating multiple *Staphylococcus* spp. with high sensitivity and specificity, even when phenotypic traits are ambiguous in bovine mastitis [37]. *S. aureus* remains a principal pathogen responsible for intramammary infections in cows and is frequently isolated from milk in both clinical and subclinical mastitis cases, whereas, in the present study, CNS has emerged as the major entity associated with bovine mastitis, emphasizing the need to reconsider their epidemiological and clinical significance. The involvement of diverse CNS species in bovine mastitis and their accurate identification using genotypic or molecular methods is, therefore, essential [38,39].

*Staphylococcus* spp. harboring *mecA* was observed in five HHs in milk, milking machine, and human samples. Methicillin resistance can be disseminated to humans by direct and indirect contact with animal or their products, and vice versa, and this concern was supported by studies showing MRSA transmission in dairy settings [40]. The study results suggested the transmission risk of AMR in the organized dairy sector, probably because of availability of very good veterinary healthcare, repeated use of antibiotics for the treatment of mastitis, and frequently changing the antibiotics in non-responsive animals. Hence, a strategy for on-farm SCM detection by CMT and early treatment with sensitive antibiotics or use of alternative plant-based antibacterial products may prevent the occurrence of clinical mastitis and AMR.

The study underscores a notable occurrence of methicillin resistance in cattle milk, with *Staphylococcus epidermidis* emerging as the predominant species. Methicillin resistance was more prevalent among associated personnel (15%) than cattle, likely due to their close interaction with the animals and the shared ecological microbiome and resistome. Reports suggest that methicillin resistance can be transmitted to humans through direct contact with animals, environmental contamination, or handling of products from infected animals [41,42]. The study limitations include, first, that the findings of the study cannot be generalized as the study was confined to limited geographic areas in Karnataka and Assam. Second, the field unit at Assam is located about 3000 km away from the ICAR-NIVEDI, Bengaluru, India, posing logistic challenges in sample collection, processing, and limiting the recovery of only *Staphylococcus* spp. Third, the interpretation of subclinical mastitis and clinical mastitis was based on CMT only and not based on physical examination of the udder because a large number of animals were recruited for the study.

Some key highlights of the study include the investigation of a total of 391 households with structured data providing a comprehensive snapshot of mastitis and AMR in the dairy industry. The Government of India has initiated a National Action Plan on Antimicrobial Resistance (NAP-AMR) to enable nationwide surveillance and to implement policies addressing this challenge [43,44]. Currently, antimicrobial treatment is indispensable to keep animal welfare and economic aspects in balance. Given that the transmission of resistant genes involves a dynamic interplay between animals, humans, and their environment, it is imperative to conduct regular surveillance of the resistance status of *S. aureus* and CoNS. This is essential to curb the spread of resistance and mitigate the disease burden associated with these resistant pathogens. Similarly, the training for farm personal on the importance of farm hygiene, clean milk production, good management, and bio-security measurements should be implemented.

## 4. Material and Methods

### 4.1. Study Site Details

The study was conducted at the organized dairy sector located in the southern state (Karnataka) and the unorganized dairy sector in the northeastern state (Assam). In the organized dairy sector, dairy is a household-based operation and farmers maintain 3–5 dairy animals in the house. There are 100–120 households in an epiunit, where farmers maintain mostly fewer than 5 cattle for milk production for economic sustenance for their families. The milk producers’ cooperative societies (MPCS) provide veterinary consultation at the farmer’s doorstep on a day-to-day basis as well as feeding, management, and husbandry protocols. The mastitis animals are tested and treated as and when reported by the farmer at their doorstep. In the unorganized dairy sector, farmers keep 30–50 milking cows in each HH. These are high-density units located in a very close proximity and routinely use feed and water from same source [45,46]. The veterinary consultation is sought as and when needed.

### 4.2. Study Design

**Figure 4:**
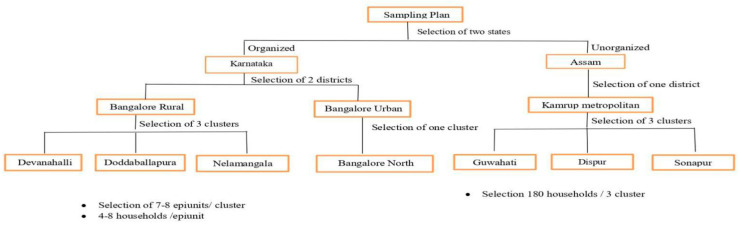
Flow chart representing study sites for sample collection.

A cross-sectional study was carried out from June 2018 to August 2019 to determine mastitis prevalence and *Staphylococcus* spp. resistance between dairy animals and associated personnel in both organized and unorganized dairy sectors. The sampling plan was designed based on a 30-cluster sampling methodology with stratification wherein the first selection was districts, followed by blocks and epiunits [45]. In Karnataka, in the first strata, two districts (Bengaluru urban and rural districts) were selected based on high milk-producing data from the Karnataka Milk Federation (KMF). In the second strata, four blocks located 25–30 km apart were identified. In the third strata, 30 epiunits located 10 km apart were selected from the list of 939 villages (7–8 epiunits/block). Finally, 211 HHs (7 HHs/epiunit) were identified based on the milking data obtained from MPCS. Sample collection in the unorganized dairy sector adhered to cluster sampling, whereas only the semi-urban district was selected as having the highest number of dairy herds. Within this district, instead of four blocks, three blocks were chosen based on areas known for high milk production. Within blocks, epiunits (termed as sub-blocks) were earmarked as follows: (a) Guwahati (8th Mile), (b) Dispur (consisting of 9th and 10th Mile), and (c) Sonapur (under the jurisdiction of Jorabat). Subsequently, 180 HHs were randomly selected (Figure 4).

### 4.3. California Mastitis Test (CMT)

After cleaning the teats, milk samples were collected, with the initial streams discarded, followed by another round of teat cleaning using cotton moistened with 70% alcohol. CMT was conducted as per the manufacturer’s protocol (DeLaval, Kansas City, MO, USA) by adding 2 mL each of milk and CMT reagent into the mastoid paddles containing four shallow cups, labeled as LF (left-fore), LH (left-hind), RF (right-fore), and RH (right-hind), and mixed well. The results were considered positive if the milk showed coagulation and change in color from white to blue in any paddle cup within 10 to 30 s. The CMT test results are graded as negative, trace, weak positive, positive, and strong positive based on visual reaction noted in the milk (-, +, ++, +++, and ++++) [4]. The trace and weak CMT positives were declared as animals with SCM, and CMT positive and strong positive milk samples were declared as animals with CM.

### 4.4. Sample Collection

**Figure 5:**
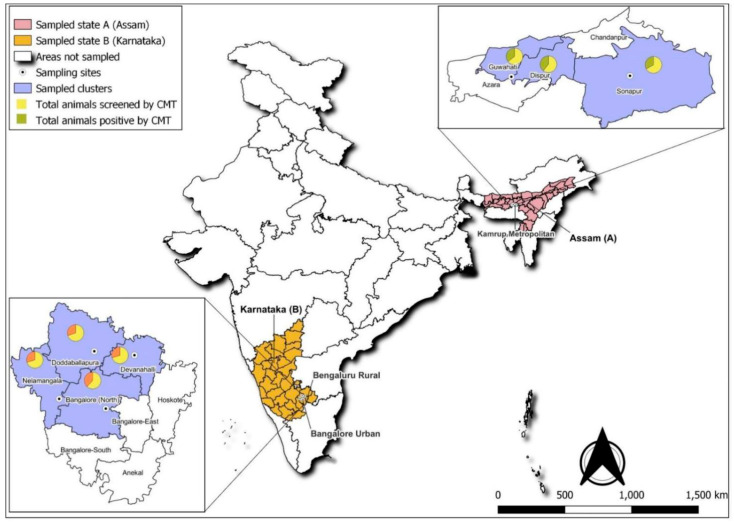
India map showing sample collection sites. A. Assam in the northeast (pink) showing sample collection sites in Kamrup Metropolitan (represented by a small circle with a dot). B. Karnataka (orange) showing sample collection sites in Bangalore rural and Bangalore north.

In the organized sector, a total of 489 samples were collected from 211 dairy HHs from four blocks of Bengaluru, Karnataka, India (Devanahalli, Doddaballapur, Nelamangala, and Bengaluru North). The samples included were pooled cow milk samples (244), associated personnel hand (109), nasal swabs (109), and milking machine swabs (27). Similarly, a total of 902 samples were collected from 180 HHs from three blocks from Assam (Guwahati, Dispur, and Sonapur). The highest number of samples were milk samples (758), followed by associated personnel hand (76) and nasal swabs (68) from Assam (Table 1 and Figure 5). In the Assam region, farmers resort to hand milking; hence, milking machine samples could not be collected. Representative four-quarter pooled milk samples of both CMT-positive (including both SCM and CM animals) and -negative animal samples were collected in sterile containers for *Staphylococcus* spp. isolation. Primarily, only pooled samples were used for downstream processing because of the limited logistical support for large-scale screening. The hand and nasal swabs from associated personnel, as well as milking machine swabs, were directly collected into brain heart infusion (BHI) broth. All the samples were transported to the laboratory in temperature-controlled sample collection boxes within four hours for further processing.

### 4.5. Isolation and Identification of Staphylococcus spp.

Milk samples (100 μL) were inoculated into brain heart infusion broth and incubated overnight at 37 °C. After incubation, broth-enriched milk samples were streaked onto selective differential mannitol salt agar (MSA, Himedia, Mumbai, India) and incubated at 37 °C for 24–48 h. This method is consistently employed for the isolation of staphylococci from bovine mastitis milk in our laboratory [35]. To obtain pure cultures, both golden yellow characteristic *S. aureus* and pink colonies other than *S. aureus* were sub-cultured onto brain heart infusion agar twice (BHI agar, Himedia, India). Based on colony morphology, catalase test, and Gram staining, *S. aureus* colonies were presumptively identified following standard protocols [47]. Phenotypically identified *Staphylococcus* spp. isolates were further confirmed by mPCR and preserved in 12% glycerol stock at −20 °C until further use.

### 4.6. Genotypic Identification and Resistant Gene Determination

**Figure 6:**
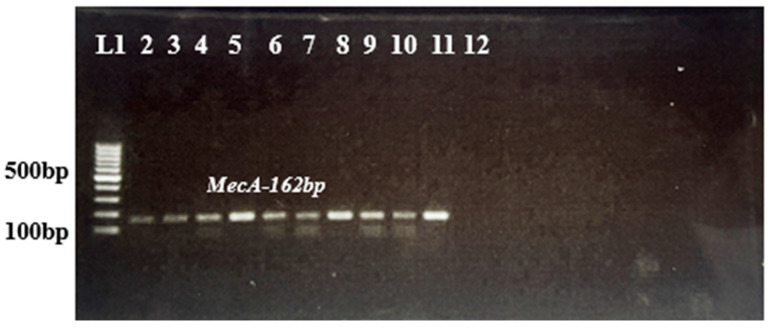
Lane 1: 100 bp ladder, Lane 2–10: *mecA*-positive (162 bp), Lane 11: ATCC 700699-positive. Control for *mecA*, Lane 12: negative control.

The preserved cultures along with two reference strains were revived initially in BHI followed by BHA. The two loops full of pure freshly grown culture for 24–48 h were used for DNA extraction (DNeasy kit as per the manufacturer protocol, Qiagen, Hilden, Germany). The quality and quantity of the extracted DNA was evaluated using a NanoDrop2000 (Thermo Scientific, Waltham, MA, USA) and also by running on 0.8% agarose gel electrophoresis. After normalizing the DNA concentrations, the mPCR was conducted to detect five major *Staphylococcus* spp. (*S. epidermidis*, *S. sciuri*, *S. chromogenes*, *S. haemolyticus*, and *S. aureus*) and methicillin-resistant determinants (*mecA* and *mecC* genes) as per the protocols described earlier [35,37]. Briefly, the 25 μL reaction volume was set using a commercially available PCR master mix, a combination of 5 sets pf primers at 0.5 μM along with 200 ng of template DNA from *S. aureus*, *S. chromogenes*, *S. epidermidis, S. sciuri*, and *S. haemolyticus* [40]. Reaction mixtures were thermally cycled once at 94 °C for 5 min, followed by 30 times at 94 °C for 30 s; 60 °C for 30 s; 72 °C for 45 s; and then once at 72 °C for 10 min. The amplified products were analyzed onto 1.5% agarose gel containing ethidium bromide (10 g mL) (Figure 6).

### 4.7. Antimicrobial Susceptibility Testing

Antimicrobial susceptibility testing (AST) was carried out in an automated system with PMIC-70 panels of 86 wells containing different antimicrobial agents (BD phoenix M50, Franklin Lakes, NJ, USA). The *Staphylococcus* spp. cultures grown on BHI agar plates overnight were inoculated into the ID broth (BD Phoenix, USA) and checked using a Phoenix Spec Nephelometer (Hanover Park, IL, USA) to set the concentration of 0.5 McFarland. As per the manufacturer’s protocol, the panels were prepared, labeled, and loaded into the Phoenix system. The results were interpreted after 16 h of incubation using Epicenter data management software version 7.22A (BD Diagnostic Systems), and the obtained result data were transferred into Microsoft Excel 2010 for further analysis. The ATCC 29213 (methicillin-sensitive) and ATCC 700699 (methicillin-resistant) strains were used as controls for antimicrobial assays.

### 4.8. Statistical Analysis

The significant difference in the mastitis detection between the states and blocks was determined by the chi-square test and *p* value using MedCalc-22.030 (https://www.medcalc.org/). The proportionate positivity at 95% confidence interval (CI) was calculated using https://sample-size.net/. Staphylococci were considered methicillin-resistant genotypically when the isolates were found to harbor the *mecA* or *mecC* gene by PCR. Maps were created using QGIS 3.34.8 software.

## 5. Conclusions

Comparative analysis between the two dairy sectors revealed a high prevalence of mastitis in Assam’s unorganized dairy sector due to overcrowding and inadequate livestock healthcare. Compared to animals, associated personnel were identified as the reservoir of resistant pathogens, suggesting probably bidirectional transmission between humans and animals. Of particular concern is the emergence of MRCoNS over MRSA strains, posing a substantial risk to mastitis in animal and public health concern. Therefore, continuous monitoring of mastitis and drug resistance profiles is essential for the development of effective treatment strategies to mitigate the mastitis burden and, thereby, antimicrobial resistance.

## Figures and Tables

**Table 1:** Mastitis detection across study states, blocks, and households.

Study State	Chi-Square/*p* Value Between Sectors	Study Blocks	Chi-Square/*p* Value Between Blocks	Households (HH)	No. of Animals Tested by CMT	CMT Positives	CMT Positivity (95% CI)
Organized sector	12.145/0.0004	Devanahalli	11.064/0.011	67	186	79	42.47 (35.27–49.92)
		Doddaballapura		46	102	44	43.14 (33.37–53.32)
		Nelamangala		56	156	66	42.31 (34.45–50.47)
		Bangalore North		42	89	55	61.8 (50.89–71.9)
		**Total**		**211**	**533**	**244**	**45.78** **(41.49–50.12)**
Unorganized sector		Guwahati	15.984/0.0003	71	668	368	55.09 (51.23–58.91)
		Dispur		32	229	149	65.07 (58.51–71.23)
		Sonapur		77	490	241	49.18 (44.67–53.71)
		**Total**		**180**	**1387**	**758**	**54.65** **(51.99–57.29)**
**Grand total**				**391**	**1920**	**1002**	**52.19** **(49.93–54.44)**

**Table 2:** Antibiotic sensitivity profiles across the study sites.

Antibiotic Groups	Percentage of Resistance (%)	
	Karnataka (Organized Sector)	Assam (Unorganized Sector)
Β—lactams (Penicillins and cephalosporins)	71.4	76.6
Lincosamide (Clindamycin)	11.5	26.8
Macrolides (Erythromycin)	13	25.1
Aminoglycosides (Gentamicin)	13	20
Sulfonamides (Sulfamethoxazole/ Trimethoprim)	10.1	14.5
Tetracyclines	9.3	13.6
Streptogramins	4.6	9.4
Quinolones (Levofloxacin)	6.6	7.7
Rifamycin	0.7	5.1
Oxazolidines (Linezolid)	0.7	5.1
Nitrofurans (Ampicillin and Cefoxitin)	3.5	3

**Table 3:** Geographic distribution of *mecA*-positive *Staphylococcus* spp. isolated from milk samples.

Geographic Location	*S. aureus*	*S. chromogenes*	*S. epidermidis*	*S. sciuri*	*S. haemolyticus*	*S. hominis*	*S. saprophyticus*	Total
**Karnataka** **(Organized Sector)**								
Devanahalli	4/35 (11.42)	0/14 (0)	6/36 (16.66)	0/10 (0)	0/2 (0)	-	-	10/97 (10.3)
Doddaballapura	7/27 (25.92)	0/6 (0)	8/25 (32)	0/1 (0)	-	2/2 (100)	1/1 (100)	18/62 (29.03)
Nelamangala	3/26 (11.53)	0/11 (0)	3/23 (13.04)	1/4 (25)	-	-	-	7/64 (10.93)
Bangalore North	2/24 (8.33)	2/13 (15.38)	7/29 (24.13)	0/1 (0)	4/7 (57.14)	-	-	15/74 (20.27)
**Total**	**16/112** **(14.28)**	**2/44** **(4.54)**	**24/113** **(21.23)**	**1/16** **(6.25)**	**4/9** **(44.44)**	**2/2** **(100)**	**1/1** **(100)**	**50/297** **(16.83)**
**Assam** **(Unorganized Sector)**								
Guwahati	3/13 (23.07)	-	0/4 (0)	0/3 (0)	2/2 (100)	1/3 (33.33)	0/1 (0)	6/26 (23.07)
Dispur	1/1 (100)	-	1/3 (33.33)	-	2/3 (66.66)	2/2 (100)	-	6/9 (66.66)
Sonapur	1/6 (16.66)	-	0/4 (0)	-	1/1 (100)	0/1 (0)	-	2/12 (16.66)
**Total**	**5/20** **(25)**	**-**	**1/11 (9.09)**	**0/3 (0)**	**5/6 (83.33)**	**3/6 (50)**	**0/1 (0)**	**14/47** **(29.78)**
**Grand total**	**21/132** **(15.9)**	**2/44** **(4.54)**	**25/124** **(20.16)**	**1/19** **(5.26)**	**9/15** **(60)**	**5/8** **(62.5)**	**1/2** **(50)**	**64/344** **(18.6)**

**Table 4:** CMT grade of milk samples and proportion of *mecA* positives.

CMT Grade	Karnataka (Organized Dairy Sector—533)	% *mecA* Positives (95% CI) *	Assam (Unorganized Dairy Sector—1387)	% *mecA* Positives (95% CI)
CMT Positives				
Trace (+)	1/17	5.88 (0.15–28.96)	2/110	1.82 (0.22–6.41)
Weak Positive (++)	4/68	5.88 (1.63–14.38)	3/186	1.61 (0.33–4.64)
Positive (+++)	9/148	6.08 (2.82–11.23)	5/297	1.68 (0.55–3.88)
Strong Positive (++++)	0/11	0.00 (0.00–28.49)	5/165	3.03 (0.99–6.93)
Total	**14/244**	**5.74** **(3.17–9.44)**	**15/758**	**1.98** **(1.11–3.24)**
CMT Negative	6/289	2.08 (0.77–4.46)	3/629	0.48 (0.10–1.39)

* CI, Confidence interval.

**Table 5:** Genotypic detection of methicillin-resistant *Staphylococcus* spp. from different sources.

	Organized Sector					Unorganized Sector							Chi-Square/*p* Value Between *mecA* Positives in Sectors
*Staphylococcus* spp.	Distribution of Samples				Total *Staphylococcus* spp. (n-454)	*Staphylococcus* spp.	Distribution of Samples			*Staphylococcus* spp. (235)			
	Milk Samples (n-241)	Milking Machine Swabs (n-22)	Hand Swabs (n-98)	Nasal Swabs (n-93)			Milk Samples (n-139)	Hand Swabs (n-40)	Nasal Swabs (n-56)		Animal Origin (Milk)	Human Origin (Hand and Nasal Swabs)	
*S. aureus* (MRSA)	13	0	2	1	16	*S. aureus* (MRSA)	2	0	0	2	*S. aureus* (MRSA) = (3.95%, 15/380)	*S. aureus* (MRSA) = (1.05%, 3/287)	
*S. chromogenes*	1	0	2	0	3	*S. chromogenes*	-	-	-	-	MRCoNS = (6.05%, 23/380)	MRCoNS = (14.63%, 42/287)	
*S. epidermidis*	2	1	6	15	24	*S. epidermidis*	8	1	9	18			
*S. sciuri*	0	0	1	0	1	*S. sciuri*	2	0	0	2			
*S. haemolyticus*	2	0	2	0	4	*S. haemolyticus*	6	2	3	11			
*S. hominis*	1	0	1	0	2	*S. hominis*	-	-	-	-			
*S. saprophyticus*	1	0	0	0	1	*S. saprophyticus*	-	-	-	-			
Total *mecA* positives	20 (8.3%)	1 (4.55%)	14 (14.29%)	16 (17.20%)	51 (11.23%)	Total *mecA* positives	18 (12.95%)	3 (7.50%)	12 (51.43%)	33 (14.04%)			
Animal origin *mecA*-positive *Staphylococcus* spp.	20/241 (8.36%)					18/139 (12.94%)							2.119/0.145
Human origin *mecA*-positive *Staphylococcus* spp.	30/191 (15.70%)					15/96 (15.96%)							0.024/0.877
Chi-square/*p* value between animal and human origin	5.013/0.025					0.337/0.562							-

## Data Availability

Upon reasonable request, the corresponding author (Rajeswari Shome) can provide the data required supporting the study.
